# Supplementary material for: Are carbon emissions trading and green financial instruments synergistic? -Comprehensive quantitative research based on content analysis
Source: PLoS One. 2024 Mar 7;19(3):e0298601. doi: 10.1371/journal.pone.0298601 (PMC10919601; doi:10.1371/journal.pone.0298601)
Supplement: S1 Data — (ZIP) [file pone.0298601.s001.zip › Data/地级市Z_F工作报告.zip!/║■─╧/│ú╡┬╩╨/└┤╫╘╣1⁄2╓┌║┼í╛┬φ┐╦╩2╛▌═°í┐_2014.docx]

各位代表：

现在，我代表市人民政府，向大会报告政府工作，请予审议，请各位政协委员和其他列席人员提出意见。

一、客观总结，去年工作成绩来之不易

2013年，我们的工作是在宏观环境错综复杂、全市经济开局不利的严峻形势下展开的，全市人民在市委的坚强领导下，凝心聚力，奋发有为，实现了经济社会发展稳中有进、稳中向好，较好地完成了预期目标任务。全年完成地区生产总值2255亿元(预计数，下同)，比上年增长10.5%；固定资产投资1300亿元，增长35%；财政总收入175亿元(含地方教育附加)，增长13.5%；城镇居民人均可支配收入22285元，增长10%；农民人均可支配收入8100元，增长13.1%。全市各项工作取得了来之不易的可喜成绩，新常德新创业的宏伟事业实现了良好开局，621万常德人民在全面建成小康社会道路上迈出了新的坚实步伐。

一年来，我们主要做了以下工作：

1、把稳增长作为当务之急

去年一季度，我市主要经济指标全面下滑，增速为近15年来最低。针对这一情况，市委市政府及时研判，果断决策，实施了稳增长的“十大举措”，扭转了下滑态势，全年增长保持在较高水平。

一是狠抓产业稳增长。把产业项目建设和实体经济发展摆在重中之重的位置。围绕壮大工业产业，重点实施了推进新型工业化的“1115”工程，对纳入“1115”工程的企业实行特殊服务、特殊政策、特殊奖励，打造常德工业的脊梁。加大园区改革与建设力度，支持区县市标准化厂房建设，实施扶持中小微企业发展的系列措施。全市完成规模工业增加值890亿元、增长11.5%，新增规模工业企业103家、总数达到897家，开工建设标准化厂房100万平方米。常德烟厂易地技扩改项目全面推进，千亿工业走廊建设加快，恒安纸业扩建、常德烟机技改、雨润食品产业园等项目进展顺利。围绕壮大农业产业，突出五个“双百亿产业”建设，促进农产品加工转化，加快现代农业示范区建设，发展新型经营主体，搞活农产品流通，扩大农村土地有序流转。战胜了严重的干旱和秋汛灾害，粮食生产保持稳定；新增规模农产品加工企业30家，新增“三品一标”认证138个，其中国家地理标志保护产品和证明商标4个；新增农民合作社420家。围绕壮大第三产业，积极打造旅游核心品牌，促进商贸流通，创新金融服务。世外桃源古镇、柳叶湖梦幻桃花岛、太阳山综合开发等旅游项目取得实质进展；居和嘉美二期、隆腾物流园等商贸物流项目建成营业；民生银行常德分行、广发银行常德分行先后开业，新增5家小额贷款公司、2家融资性担保公司。

二是扩大内需稳增长。扩大有效投入，集中精力加快交通、水利、能源等重大基础设施建设，实施一批重大产业、民生、环保项目。年初确定的500个重点工程项目，完成投资668亿元，其中268个竣工投产；500个重大前期项目，有225个提前开工建设。投资结构进一步改善，民间投资占比达到65%，工业、基础设施、民生投资分别增长37%、30%、40%。扩大居民消费，培育消费热点，实现社会消费品零售总额725亿元，增长13.5%。

三是搞活要素稳增长。扩大和搞活资金融通。全年争取上级投资和项目资金210亿元，增长10%；大力招商引资，引进内外资金470亿元，增长30%；完善中小企业融资担保体系，市财鑫担保公司为各类创业主体提供融资服务52亿元；鼓励金融机构扩大信贷投放，各项贷款余额801亿元，新增160亿元。保障和规范用地需求。市政府新成立了由市长任主任的土地管理委员会，在土地规划、储备、供应等方面实行“十统一”的管理，从严规范土地市场，切实保障大项目、好项目用地需求，全年批回建设用地2.5万亩。充分发挥科技和人才效能。实施省级以上科技项目78项，新增产学研合作项目94项，新认定高新技术企业19家，高新技术产品产值达到550亿元；为企业输送急需人才1500多名，全市高技能人才总量达到4.9万人。

2、把谋长远作为创业之要

以不负一方山水、不负一方人民的忠诚情怀，以敢于担当、勇于创业的开拓精神，市委市政府科学谋划常德的长远发展，全面运筹发展的整体布局，响亮地提出了“新常德新创业”的总要求，描绘了“一个中心、两个转变、三个翻番、四个常德”的宏伟蓝图。

一是在重大空间规划上谋长远。展开了中心城区500平方公里高速环圈内空间规划的研究。启动了常德市总体城市设计，推进中心城区和周边区域的水系、生态保护等专项规划编制。谋划推进中心城区“四张大门”、“两条轴线”建设。

二是在重大产业项目上谋长远。千方百计争取中联重卡、中国南车、忠旺集团、中国移动湖南数据中心等具有战略意义的项目引进，推动芙蓉王现代新城、现代农业十大标志性工程、旅游六大精品工程建设，以形成多点给力、多元支撑的产业发展格局。

三是在重大基础设施上谋长远。谋划“一纵一横”铁路网络，“一环六射”、“一纵一横”高速公路网络，市域“四纵三横”沅澧快速干线网络，构建周边城市2小时通勤圈、市域内1小时经济圈。积极运作宜张高速、宜常客运专线等重大项目。

四是在重大改革事项上谋长远。对市委确定的“十项改革”任务，明确专门班子负责。谋划和制定加快全面建成小康社会、常德经开区二次创业、现代农业示范区建设、口岸建设等方面的政策措施。

3、把抓城建作为开篇之作

为了更有效地落实国家加强城市基础设施建设的政策措施，为了让全国文明城市的称号更加名至实归，为了更好地发挥城市的承载功能，为了让生活在这座城市的人民更有归属感、幸福感和自豪感，强有力地推进了城市的建设和管理工作。

一是强力推进城市建设“三改四化”。把中心城区道路改造这一良心工程、民生工程作为新常德新创业的开篇之作，组织了以杆线入地、地下管网改造、通行能力提高、公交专用道建设、慢行系统构建、路面提质改造为主要内容的路改大会战，207条、157公里、247万平方米的路改主体工程基本完成。调动多方力量，采取多种举措，从严监管工程质量、施工安全、交通组织、工程廉洁等环节，路改的效果已经显现。一批水改综合治理项目开工建设。改造城市棚户区10419套。武陵大道等重点区域的美化、亮化即将完成。新增绿地100万平方米，栽植乔木3万株。智慧城管、智能交通、平安常德三大数字化平台建设正在实施。路网建设同步推进，桃花源大桥建成通车，促进了“一江两岸”一体发展。

二是积极推进城市管理“一改四化”。着手实施市城区城市管理体制改革，探索建立运营市场化、管理网格化、作业精细化、考核标准化的新机制。开展完美社区建设前期工作，社区服务和管理能力有新的提升。顺利通过全国文明城市公共文明指数测评。

三是全面推进县(市)城区提质扩容。各县(市)进一步完善城乡规划体系，组织实施一大批基础设施和功能性项目。桃源县城成为国家智慧城市试点镇，津澧融城进入省级规划层面，汉寿大道、安乡大道建成通车，临澧县城滨河新区、石门县城东城新区建设加快。全市城镇化率达到44.5%，提高1.5个百分点。

4、把强基础作为发展之先

一是破解交通瓶颈。加强汇报衔接，加大资金投入，加紧调度协调，加快建设进度。岳常高速建成通车，东常高速完成主体工程，安慈高速获批立项。新建改建干线公路68公里，汉寿县城至太子庙、澧县黄桥至垱市公路建成，319国道桃花源景区改线、周家店至西湖、西湖园艺村至汉寿罐头嘴等公路开工建设，官垱大桥、陆家渡大桥重建等工程开工，安乡交通“孤岛”问题正在得到解决。石长铁路增建二线工程加快建设，黔张常铁路通过国家发改委可研评审。桃花源机场扩建顺利推进。盐关码头获批建设国家二类口岸，德山港区等7个千吨级码头获批立项。建成农村公路397公里，实施病危桥改造149座，澧水张公庙大桥、桃源白洋河大桥、石门三江口大桥完成改造。

二是完善水利设施。投资24.5亿元，建设各类水利工程3.5万处，新解决了57.5万农村人口饮水安全问题，湖区蓄洪垸堤防加固、主要支流治理、小农水重点县建设、山洪灾害防治等项目取得明显成效，获得全省水利建设“芙蓉杯”。实施环洞庭湖基本农田建设工程。建成高标准农田和改造中低产田15万亩。

三是推动能源建设。历经十年不懈努力，常德电厂通过国家发改委核准。桃源水电站首台机组并网发电。建成了一批输变电工程，完成了228个行政村的农网升级改造。

四是促进“两型”发展。狠抓节能减排，严控高能耗、高污染项目上马，实施重点节能项目22个、重点减排项目93个，完成了节能减排约束性指标。加大生态环境保护力度，推进河流、湖泊、土壤生态修复，沅澧两水水质持续保持III类以上标准，西洞庭湖保护区获批国家级自然保护区。加强生态市建设，通过国家环境保护模范城市复核，启动国家森林城市创建工作。开展农村环境集中整治，推进垃圾分类处置，巩固水库禁止投肥养殖成果，启动了珊珀湖、北民湖、西毛里湖等“万亩湖泊”的禁止投肥养殖，III类以上水质的水库达到91.7%。新造林27.1万亩，封山育林18万亩。

5、把惠民生作为施政之本

我们坚持民生优先，全面完成了省为民办实事、市八大重点民生工程建设各项指标任务。全年用于民生的财政支出超过70%。

就业和社会保障得到加强。开展就业培训8.5万人，新增城镇就业7万人，新增农村劳动力转移就业9.4万人。五类社会保险新增参保9.6万人次。企业退休人员基本养老金、城乡低保补助水平、农村五保供养标准全面得到了提高。改扩建农村敬老院18所，市第二福利院等示范养老机构建设进展顺利。儿童福利和残疾人服务体系建设加快，我市被确定为全国未成年人社会保护试点城市。

扶贫攻坚取得新的进展。投入2.4亿元，对160个扶贫点村的基础设施进行改造，农民生产生活条件得到改善。推动产业扶贫、项目扶贫，加大对扶贫帮困对象的支持扶助力度。年内有11.3万人实现脱贫。

教育发展水平进一步提高。促进义务教育均衡发展，合格学校建设、校安工程、课桌椅配套工程任务较好完成，学前教育普惠工程扎实推进，高中教育质量进一步巩固，高考成绩综合排名位居全省第二。湖南幼师高专挂牌成立，常德技师学院筹建、同德职院“专升本”取得积极进展。学校文化建设加强。

医疗卫生服务能力继续增强。全市新农合参合461.2万人，参合率达到99.4%。医药卫生体制改革稳步推进，公共卫生服务项目全面实施。重大传染病防控有力，各类突发公共卫生事件处置有效。市第一人民医院扩建、湘雅常德医院新建等一批项目进展顺利。加强人口计生工作，基层基础进一步夯实。

文化建设呈现生动局面。继续推进广播电视村村通村村响、城乡公益电影放映、送戏送书下乡等文化惠民工程，“百团大赛”等群众文化活动蓬勃开展。城头山纳入国家考古遗址公园立项名单，全国首家微型小说创作基地落户武陵区，常德鼓书和市京剧协会艺术团进京演出获得成功。文化产业发展态势良好，文化市场监管有效。新增国家重点文物保护单位22家。

其他各项事业协调发展。成功举办市第六届运动会，参加第十二届全运会取得好成绩。与斯里兰卡阿努拉达普拉市缔结为友好城市。审计、统计、粮食、物价、人防、气象、地震、档案、民族宗教、对台、方志、科协、贸促会、红十字会等工作取得新成绩。工会、共青团、妇联在经济社会发展全局中发挥了重要作用。

国防教育、国防动员、国防后备力量建设加强，“双带双促”、“双拥”共建活动深入开展，全省“双带双促”现场会在我市召开。

6、把优环境作为革命之举

我们把优化经济发展环境作为一场革命来进行，革自己的命、削自己的权、去自己的利，其目的就是要把常德打造成全省审批最少、流程最短、收费最低、服务最优的城市。

一是打造宽松高效的政务环境。以“六个压减”为抓手，促进政务服务提速提质。市本级行政审批项目压减了41%，行政审批时限压减了64%，考核评比项目压减了50%以上，奖励项目压减了83%、奖励金额压减了50%，市政府各类领导小组压减了65%，市一级行政机关会议费、学习考察费、公务接待费压减了25.8%、67.5%、16.8%。

二是打造规范有序的建设环境。建立企业和项目周边环境事件快速处置、责任追究机制。对强行参工参运、串标围标、欺行霸市、违法建设等行为，予以重拳出击、集中治理，共处置相关案件100多起，刑事拘留37人。

三是打造安宁和谐的社会环境。推进平安常德建设，实行社会稳定风险评估，开展“三打一整”专项行动，加强了对流动人口、特殊人群的管理服务，社会治安形势稳定。推进涉法涉诉信访改革，强化领导干部接访下访、部门联合接访和基层基础建设，信访热点难点问题得到较好化解。持续开展打非治违、隐患排查专项整治，集中开展安全生产大检查，防止了重特大事故发生。加强食品药品监管，实施食用油放心工程，没有发生重大原发性药品和群体性食品安全事件。应急处置、事故救援和抗灾救灾能力进一步增强。

过去的一年，我们认真抓好政府自身建设，努力建设创新型政府、法治型政府、服务型政府、实干型政府、廉洁型政府。切实转变政府职能，坚持依法行政，推行政务公开，强化行政问责，落实中央、省委、市委关于改进工作作风、密切联系群众的各项规定，坚持不懈开展反腐败斗争。我们全面落实市委的决策部署，认真执行市人大及其常委会的决议决定，自觉接受市人大及其常委会的法律监督和工作监督，自觉接受市政协的民主监督，认真办理人大代表建议、政协委员提案，重视社情民意处理，积极采纳参政议政成果，政府的权力在阳光下运行，施政行为经受了实践和群众的检验。

各位代表!过去的一年，常德发展翻开了新的一页，开启了新的征程。我们取得的每一点成绩，实现的每一点进步，都是市委统揽全局、正确领导的结果，是市人大、市政协加强监督、全力支持的结果，是全市人民万众一心、奋力拼搏的结果。在此，我代表市人民政府，向辛勤工作在各条战线、各个岗位上的全市人民，向各位人大代表和政协委员，向各民主党派、工商联和社会各界人士，向驻常人民解放军、武警消防官兵和公安政法干警，向所有关心、支持常德发展的同志们、朋友们，表示衷心的感谢!

二、冷静分析，今年发展任务尤为艰巨

2014年，是全面深化改革之年，是全面推进新常德新创业之年，也是全面建成小康社会和实现“十二五”规划的关键之年。当前，改革、发展、稳定面临的环境和条件都在发生深刻的变化。

我们应该看到，常德正处于攻坚克难的关键期。改革发展仍然面临不少困难和挑战，政府工作任务异常艰巨。经济总量不大，人均均量和发展质量不高的状况还没有根本改变；产业结构不合理，特别是工业项目带动力不强的状况还没有根本改变；交通、水利设施建设仍然滞后，资金、人才等瓶颈制约突出的状况还没有根本改变；城镇化水平偏低，县域经济比较薄弱的状况还没有根本改变；保障和改善民生任务艰巨，影响社会和谐的不确定性因素较多的状况还没有根本改变；市场秩序不规范，经济发展环境不优的状况还没有根本改变。同时，政府职能转变与全面深化改革的要求还不适应，政府工作人员的思想观念、工作作风与实践党的群众路线的要求还不适应，形式主义、官僚主义、享乐主义和奢靡之风在一些地方和部门仍然存在。

我们更应该看到，常德正处于大有作为的“黄金期”。我们实现赶超发展，正逢其时。有好机遇：党的十八届三中全会全面深化改革的总体部署，为我们带来了最大的发展红利；中央坚持稳中求进的工作总基调，使我们的发展处在一个稳定向好的宏观环境；国家实施长江经济带发展战略，重视洞庭湖生态经济区建设，有利于我们更好地发挥地处“过渡带”、“结合部”的区位特点和优势，在基础设施建设、产业布局等方面争取国家更多的支持。有好基础：经过多年努力，全市经济具备较强实力，交通体系较为完备、日益改善，城市承载能力、产业扩张能力正在提升。有好平台：常德好山好水好地方，是一块创业创富的热土，是一个干事成事的福地。有好来势：新常德新创业的全面推进，给沅澧大地注入了蓬勃生机和巨大活力，发展的内生动力和经济的潜在能量将更好更快地释放出来。有好氛围：常德的干部群众素质高、能力强，常德的各级组织顾大局、重职守，在市委的坚强领导下，“几大家”和衷共济、团结一心，全市上下风清气正、心顺劲足，凝聚起了强大的发展正能量。

总之，2014年，机遇与挑战同在，希望与困难并存，机遇难得，希望可期。我们要时刻保持清醒头脑，既要注意问题导向，又要发扬工作成绩；既要坚持底线思维，又要追求更高目标；既要增强忧患意识，又要树立发展信心，努力把政府工作提升到更高层次、更高水平。

政府工作的总体要求是：全面贯彻党的十八大和十八届二中、三中全会精神，认真落实中央、省委、市委经济工作会议精神，把握稳中求进的工作总基调，把握新常德新创业的总要求，坚持改革创新，坚持“三量齐升”，强化基本公共服务，着力打好民生升温、园区攻坚、城市提质三大战役，促进经济持续健康发展、社会和谐稳定。

经济社会发展的主要预期目标是：地区生产总值增长10.5%以上，固定资产投资增长30%以上，规模工业增加值增长13%以上，财政总收入和公共财政预算收入增长13%以上，城镇居民和农民人均可支配收入增长13%以上，居民消费价格指数控制在全省平均涨幅以内，完成省委省政府下达的节能减排约束性指标。

按照上述总体要求，完成发展预期目标，必须努力抓好以下十个方面的工作：

1、坚持改革引领

把学习贯彻党的十八届三中全会精神作为一项重大政治任务，通过深化改革破解发展难题，通过扩大开放赢得发展机遇。

以更大的力度推进改革。按照中央统一部署，深化经济体制、政治体制、文化体制、社会体制、生态文明体制各方面改革，推进治理体系和治理能力现代化。特别是把握经济体制改革这个重点，使市场在资源配置中起决定性作用和更好发挥政府作用。扎实推进市委市政府确定的“十项改革”。投融资体制改革主要是完善政府性投融资公司法人治理结构，创新融资模式，防范和化解政府债务风险。园区管理体制改革主要是构建“一权两制一司”的管理模式。城市建设与管理体制改革主要是逐步实行重心下移、分级管理、市场运作，调动和发挥各级积极性。农村经济体制改革主要是促进要素合理流动，创新农业经营体系，完善林权质押等制度。土地管理制度改革主要是建立城乡统一的建设用地市场。积极对接中央财税体制改革，理顺市区财政体制。完善国有资产管理机制，提高国有资产管理水平和使用效益。推进事业单位分类改革，实施公路管理体制等方面改革。推进农村信用联社改制为农村商业银行。按照统一要求，搞好地方政府机构改革和职能转变。

以更大的力度扩大开放。加强开放平台建设，更好发挥海关、检验检疫等职能机构的作用，申报常德经开区公共保税仓，确保盐关码头二类水运口岸建成运营。推进与泛湘西北城市群融合，加强与长株潭、洞庭湖生态经济区和长江经济带城市的对接，开辟新的铁路、港口、航空线路。加强与国内外友好城市、港澳台地区交流，争取更多国际国内高水平赛事、展会落户常德。实施“走出去”战略，鼓励本地企业境外上市。切实降低门槛、放宽准入、激发活力，鼓励全民创业，支持和引导非公有制经济健康发展。

2、坚持工业主导

突出打好园区攻坚战役，努力实现全市规模工业增加值突破1000亿元、规模工业企业户数突破1000家的目标。

全力推进“1115”工程。实施专项规划，落实“三特”政策。加快千亿工业走廊建设，争取中联重卡、中联现代农业装备等重大项目落地。推进常德经开区二次创业，抓好项目立区、产业强区、机制活区，启动南车产业园等一批重大项目，实现生产总值、工业总产值、财政收入等主要指标30%以上增长。夯实千亿烟草产业基础，常德烟厂易地技扩改主体工程要有突破性进展，确保常德烟机技改、金鹏印务芙蓉王烟标生产线技改等项目建成投产。支持“1115”工程企业做大做强，主营业务收入过50亿元的企业增加到3家。

加快提升工业园区。抓好园区规划修编，完善园区空间布局和产业规划，促进产城融合，争创国家级高新区和经开区。改革和完善园区管理体制、运行机制，激发园区内生动力和发展活力。加强园区功能区规划建设，吸引各方面资本投入生产生活服务设施建设，全年完成园区基础建设投入25亿元以上。市财政安排1亿元资金，对各园区标准化厂房建设实行以奖代补，全年建设标准化厂房200万平方米。

积极支持中小微企业。加强财税扶持，市财政整合安排专项资金1500万元。做好金融支持，鼓励金融机构扩大信贷投放，对融资性担保机构新增中小微工业企业担保贷款给予财政风险补偿，支持企业在全国中小企业股份转让系统融资。优化涉企服务，推进工商注册制度便利化，加快市县中小企业综合服务平台建设，稳定扩大政府采购项目面向中小微企业的份额，优先采购“两型”产品。切实减轻企业负担，严控各类涉企检查，坚决取消各种不合规收费，对符合政策的企业给予培训补贴和社保补贴。引导个体工商户转型升级为企业。全年新增小微企业1000家以上。

完善延伸产业链条。实施产业升级工程，着力培育烟草、装备制造、食品生物医药、有色金属及新材料、纺织服装、电子信息等六大产业集群。增强常德烟厂、创元铝业、中联重科、云锦纺织等龙头企业的带动辐射能力。引导中小企业围绕龙头企业的上下游产品进行产业配套，提高重点产品聚集度。推进工业企业名品名牌创建，全年新增中国驰名商标3件以上。

突出抓好招商引资。着力引进战略投资者，争取忠旺集团、力元新材能源包等战略投资项目落户常德。年内全市组织3次大的专题招商活动，突出长三角、珠三角和港澳台等重点区域招商，实现承接沿海产业转移新突破。抓好以商招商、平台招商、商会招商，分产业、分行业组建专业专职招商小分队。实施招商引资“一把手”工程，党政主要负责人要发扬“蚂蟥精神”，用“钉钉子”的办法，务求招商引资的实效。全年引进投资过亿元工业项目100个以上，引进内外资500亿元以上，直接利用外资增长15%以上。

3、坚持城镇带动

突出打好城市提质战役，推进以人为核心的城镇化，加快建设泛湘西北现代化的区域中心城市。

优化空间布局。完善市域城镇体系规划，推动城市与城镇协调发展、产业与城市城镇融合发展。中心城区按照“沿江发展、滨湖生活”的空间布局，坚持内提外拓、统筹发展的理念，构建“一江两岸、一体三极”的城市发展新格局，做好城市功能分区、江北江南统筹发展、水系连通等策划规划，完成总体城市设计和生态保护、水系、景观等专项规划编制，让城市融入大自然，让居民望得见山、看得见水、记得住乡愁。

提升品质品位。进一步完善中心城区路网体系，推进洞庭大道东拓、柳叶大道西延、皂果路过江通道等骨干路网建设。进一步搞活城市经营，突出柳叶湖、白马湖、江南风光带等区域的规划建设，启动白马湖国际商务中心、常德大剧院建设，高水平打造地标性建筑。进一步推进城市建设“三改四化”，完成路改扫尾工程，加快城市和周边城镇雨污分流、江河湖水连通等工程，全市完成棚户区和城中村改造2万户以上；继续开展“城市家具”和门店招牌整建，完成主次干道沿线拆围透绿工作，搞好临江建筑物、城市桥梁、街道路灯的夜景景观设计与建设，提升数字化系统运行管理水平。全面实施城市管理“一改四化”，完善城市管理长效机制，开展中心城区交通秩序和市容环境卫生综合整治，巩固提升全国文明城市和国家卫生城市建设成果。

完善城镇体系。加强县城和小城镇发展规划，进一步优化城镇布局，推进县城撤镇并乡设街道，提高县城在县域经济中的首位度。引导各县(市)城区以宜居宜业为取向，加快推进地下管网、城市道路等基础设施建设，开工建设“五县一市”天然气输配管网工程。积极推进津澧融城，打造市域“副中心”。争取将千亿工业走廊和城镇带发展纳入全国推进新型城镇化试点。重点支持和培育一批有地域文化积淀、有持续发展潜力的特色镇。推进户籍制度改革，全面放开建制镇和小城市落户限制，推进农业转移人口市民化。全市城镇化率达到46%以上。

建设完美社区。按照“以人为本、信息化支撑、网格化管理、社会化服务”的要求，推进完美社区建设，优化调整街道、社区的布局和区划，开展“3+N”社区管理模式试点。市财政连续3年每年安排1亿元，带动各方投入3亿元以上，重点推进社区办公服务场所、警务室、卫生健康服务室、文体活动室、幼儿园等基本公共服务设施建设。

发展现代服务。以城镇为依托，大力发展旅游、金融、物流、文化创意、电子商务等现代服务业，促进传统服务业进一步提升。推进旅游产业转型升级，积极发展休闲旅游等新业态，加强旅游资源整合和宣传营销，加快创建柳叶湖国家级旅游度假区和桃花源国际文化旅游度假区，推进六大旅游精品工程，确保欢乐水世界水公园投入运营，建成世外桃源古镇一期工程，完成太阳山旅游配套设施建设。支持区县市发展特色旅游。提升金融业发展水平，优化金融生态环境，年内引进和培育村镇银行、民营银行、小额贷款公司等各类金融机构15家以上，新增银行贷款120亿元以上、表外融资120亿元以上。重视和加强企业上市工作，力争1家以上企业挂牌上市。推进城市商圈、大型专业市场、物流园区和临空产业园建设，抓好湘西北汽贸城、东鹏国际陶瓷中心、兴隆国际装饰城等项目。培育壮大影视传媒、动漫游戏、新闻出版、演艺娱乐等特色文化产业，组织好湘西北首届动漫大赛。发展电子商务、信息咨询、服务外包、健康养老等新兴服务业，促进房地产业健康发展。出台政策措施，完善配套服务，鼓励发展总部经济。全年完成社会消费品零售总额820亿元，增长13%。

4、坚持强农惠农

跳出农字抓农业，跳出农业抓农村，推进农业现代化和城乡一体化，让农业成为有奔头的产业，让农民成为很体面的职业，让农村成为美丽的家园。

壮大优势产业。落实国家粮食安全战略，推进粮食、生猪、水产、蔬菜、林业五个“双百亿产业”建设，抓好棉花、柑橘、油料、禽蛋等特色产业发展，因地制宜发展花木、茶叶、烟叶、葡萄等高效产业。健全农业技术推广、农产品检验检测体系，加大优质种苗、先进实用技术和农业机械推广力度。完善农业保险。高度重视防汛抗旱。加强动植物防疫、森林防火和农产品质量安全工作。

推行现代经营。培育新型经营主体，推进和规范农村土地流转，发展多种形式规模经营。新发展农民合作社500家以上、生产经营大户2000户以上。继续实施加工流通“双百双十”工程，加快推进雨润食品、金健米业百亿工程、常德农产品物流交易展示中心等产业化项目。完善标准体系，推进生产、加工、销售各个环节标准化。发挥好市现代农业投资开发公司的作用，提升西湖西洞庭国家现代农业示范区建设水平，推进常德农业科学院建设，加大区县市现代农业示范区建设力度。开展争创农业品牌活动，新增“三品一标”认证100个以上，其中国家地理标志保护产品和证明商标3个以上。

完善基础设施。加强水利设施建设，推进小型水库除险加固、蓄洪垸堤防加固、三峡后续影响处理、中小河流治理等项目建设，启动沅澧水河湖连通工程。推进小型农田水利建设三年行动。完善水利工程日常管理机制，确保长效运行。抓好农村公路、通信、机耕道建设，推进农网升级改造，完成德山善卷、鼎城黄土店等输变电工程。推进农业综合开发，建设高标准农田和改造中低产田12万亩。

建设美丽乡村。按照生态宜居、生产高效、生活美好、人文和谐的要求，全面启动美丽乡村建设。市财政安排专项资金2000万元。推进美丽乡村建设规划编制，开展“百村示范、千村创建”行动，探索农村社区“3+X”建设模式，发挥农民群众的主体作用，调动社会力量投入建设的积极性。重点整治农村环境，推行农村垃圾分类减量、分户处理，加强农村面源污染和畜禽集中养殖污染治理。

增加农民收入。发展观光农业、乡村旅游，支持种养能手、返乡农民工创办实业，增加经营性收入。发展劳务经济，推动农村劳动力转移，增加工资性收入。保障农民对承包地、集体资产和土地增值的收益权，增加财产资产性收入。执行国家粮食最低收购价政策，保持农民负担监管高压态势，保障各项惠农政策全面落实。

5、坚持交通先行

实施现代立体交通体系发展规划，掀起新一轮交通大投入、大建设高潮。

推进重点工程。年内常安高速，澧水艳洲大桥，澧县黄桥至卷桥、三岔路至临澧、周家店至西湖、西湖园艺村至汉寿罐头嘴等公路建成通车。加快石长铁路增建二线、桃花源机场扩建、319国道桃花源景区改线、临澧安福至石门青玄公路、临澧林伯渠故居至石门夹山公路建设，抓好陆家渡大桥和官垱大桥建设，力争完成主体工程。开工建设黔张常铁路、安慈高速、长常高速常益段改造、207和319国道市城区改线、石龟山大桥重建、石门太平至柳家垭公路等项目，加快宜张高速等项目前期工作。

培育重大项目。积极对接国家区域和交通发展规划，开发一批提升枢纽位置的重大交通项目，把更多项目挤进上级笼子，推进更多项目提前开工建设。谋划和启动长益常城际铁路、常岳九铁路、宜常客运专线、洞庭湖环湖公路、常德综合枢纽站等重大项目。

加强交通管理。加大路政执法力度，全力抓好治超工作，全面整治超限超载运输。加强公路管理养护，完善干线公路、农村公路管养机制，提高公路维修及时度和综合优良率，确保交通设施建得起、管到位、受益久。

6、坚持区域协调

以全面建成小康社会为统揽，加快发展县域经济。

强化分类指导。认真落实省委省政府、市委市政府有关分类指导县域全面建成小康社会的意见和措施，实行以县为主、分类施策。积极帮助区县市争取上级转移支付和项目资金，市财政整合安排专项资金支持县域经济发展。区县市重大产业项目优先列入市重点项目建设计划。鼓励市级金融机构在区县市设立分支机构，支持市级担保公司扩大对县域经济的担保投资。

强化市县联动。科学制定产业发展规划、城乡基础设施规划、中心城镇和中心村发展规划“三张蓝图”，积极打造推进县域经济发展的公司化平台、农业科技园区、现代农业示范区“三大平台”，加快构建市到县和县域之间的“四纵三横”快速通道。在充分论证的基础上，尽快启动沅澧快速干线一、二号大道建设。加强市县之间的项目对接。

强化自主发展。各区县市要依托产业基础和资源条件，扬长避短发展工业，抓好传统产业提质和新型产业培育；加强城镇规划布局和基础设施建设，把县城和中心镇做强做实；加快调整农业结构，发展有效益的特色种养业。争创全省县域经济十强县、二十强县。

强化基层基础。坚持重视基层、加强基层、支持基层、服务基层。完善对口帮扶制度，改善基层生产生活条件。保障乡村组织正常运转，保障基层干部正常待遇。鼓励和促进基层干部热爱基层、扎根基层，记好民情账，绘好村情图，练好服务功。组织好第九次村民委员会换届选举工作。

7、坚持科教先导

推进智慧常德建设，培育强劲的创新驱动能力、教育支撑能力、智慧发展能力。

实施科技创新计划。突出企业创新主体地位，重点建设企业研发机构、重点实验室、工程技术研究中心和教授工作站等科技创新平台，全年培育4家院士工作站。实施一批重大科技专项，推进常德智慧谷、常德技术转移中心等科技创新载体建设。促进科技与金融结合，优化科技投入机制，推进博士创新创业行动，重视科技创新人才队伍建设。加快培育高新技术企业，年内新增高新技术企业10家以上，高新技术产品产值达到650亿元。

实施教育提升计划。实施合格学校建设和校安工程，调整优化学校布局，编制中心城区学校布局规划。进一步提升普通高中教育办学水平。培育职教品牌，加强职业教育示范学校、精品特色专业建设。促进高等教育升级提质，支持同德职院“专升本”和湖南高尔夫旅游职院争创全国示范性高尔夫教学实训基地，加快湖南幼师高专、常德技师学院新校区建设，申办常德财经职业技术学院。支持学前教育、民办教育、特殊教育、社区教育、老年教育健康发展。促进教育公平，抓好家庭困难学生资助工作，完善校长教师交流轮岗制度，推进教育信息化工程，缩小区域、城乡、校际差距。创新教育激励机制，加强学校文化、师德师风和平安校园建设。

实施信息应用计划。启动常德云计算中心建设，构筑全市统一的信息资源共享交换系统，拓展提升常德政府网站群，建设面向公众、企业、政府的公共信息服务平台。管好用好智慧城管、智能交通、平安常德数字防控系统，建设社会管理综合信息平台，提升社会管理和公共服务的智能化水平。加快推进“三网融合”。发展一批信息化应用示范企业和示范园区，积极培育智慧产业。

8、坚持绿色发展

良好的生态环境是常德的最大优势和最大财富，我们要像保护眼睛一样保护好青山绿水、蓝天净土。

倡导生态理念。加强生态文明教育，开展节能减排、环境保护全民行动，落实节能、节水、环保产品消费政策，大力倡导生态环保的生产方式、生活方式和消费模式。开展生态文明家庭、学校、社区、乡镇创建活动。加强生态文明制度建设，落实最严格的源头保护、损害赔偿、责任追究制度。

改善生态环境。加快生态市建设进程，加大耕地、森林、河流、湖泊、湿地保护和修复力度。开展植树造林，巩固退耕还林和封山育林成果，抓好城镇绿荫工程和公园建设，创建国家森林城市。强化重点区域地质灾害防治。抓好大气污染、固体废物污染和城市噪声、油烟、粉尘污染专项治理，推进中心城区“煤改气”和机动车环保标识管理。

发展生态经济。加快产业转型升级，发展绿色低碳产业，坚决防止高能耗、高污染项目引进。严格落实节能减排目标责任制，抓好工业、建筑、交通等重点领域节能减排。继续推进电力、水泥、化工等重点行业节能减排工程建设，重点实施火电行业脱硫脱硝，城镇污水处理，印染、造纸等废水深度治理和涉重金属污染治理。加强资源集约节约利用，提高资源利用效率。

9、坚持民生优先

突出打好民生升温战役，全面完成省市为民办实事任务，推进幸福常德建设。

扩大城乡就业。落实政府促进就业责任，多渠道开发就业岗位，重点促进高校毕业生就业，做好农村劳动力转移就业和城镇困难人员、退役军人等就业。加强职业技能培训和创业培训指导，建设覆盖全市的公共就业人才信息服务系统，加大市场配置人力资源的推进力度。完善企业工资集体协商与劳动关系协调机制。年内新增城镇就业6万人以上，新增农村劳动力转移就业6万人以上。

完善社会保障。健全覆盖城乡的社会救助体系，建立救助申请家庭经济状况核对机制，提高城乡低保、农村五保供养水平，使特困人员不为饥寒所迫、大病所困、失业所忧、灾害所难。开展社保扩面行动，推进社会保险全覆盖，年内新增各类保险参保8万人次以上。完善社会保险关系转移接续政策，在全市推行“五险”统一征缴。办好示范养老机构，改建扩建农村敬老院15所，引导和加快社会养老服务体系建设。健全农村留守儿童、留守妇女、留守老年人关爱服务体系。推进保障性安居工程，年内建设5.2万套。发展公益慈善事业。

加强公共卫生。深化医药卫生体制改革，巩固基本药物制度，开展城乡居民大病保险试点，稳步推进公立医院改革。完善医疗卫生服务体系，加快街道卫生服务中心、社区卫生健康服务室、标准化村卫生室建设。抓好疾病防控、妇幼保健、爱国卫生运动工作。发展中医药事业。做好人口计生工作。

繁荣文化事业。统筹推进文化基础设施和服务设施建设。实施农家书屋、广播电视村村通村村响等文化惠民工程，支持文艺精品创作。发掘地方历史文化，保护利用好各类文化遗产，争创国家公共文化服务体系示范区和鼓书文化生态保护区。开展“百团大赛”等群众文化活动，创建“中国书法名城”。加强文化市场监管。

抓好安全饮水。把解决好城乡居民饮水安全问题作为政府工作的重大任务。建立工作机制，做好科学规划，强化治污措施，抓好工程建设，筹划备用水源。实行最严格的饮用水源地保护措施，强力推行水库、河湖禁止投肥养殖，中心城区周边禁止畜禽集中养殖，保护好沅水、澧水母亲河，保障饮用水源安全。全年解决50万农村人口饮水安全问题。

推进扶贫攻坚。坚持真扶贫、扶真贫，推进科学扶贫、精准扶贫。切实抓好300个重点村的扶贫帮困工作。做好发展生产、健全公共服务和教育三件大事，制定实施义务教育、危房改造、饮水安全、卫生设施、交通基础、社会保障6件扶贫实事三年行动计划，做到一家一本台帐、一个脱贫计划、一套帮扶措施，推动水、电、路、气、房和环境整治“六到户”。全年实现脱贫11万人以上。支持革命老区加快发展。搞好移民安置和后扶工作。

发展各项事业。增强财政杠杆意识，加强财税收入征管，优化财政支出结构。严格执行土地管理“十统一”规定，做好征地拆迁安置工作，促进节约集约用地，保障重大项目用地需求。加强住房公积金归集管理。加强知识产权保护。加大科普宣传力度。认真组织第三次全国经济普查。开展全民健身运动，提升竞技体育水平。完善残疾人保障和服务体系。做好审计、人防、外事侨务、民族宗教、地震、方志、档案、保密、对台、气象、驻外机构等工作。发展老龄事业，做好老干部工作。积极支持工会扩大工作覆盖面，维护职工群众切身利益。重视和支持共青团、妇女儿童工作。

统筹推进国防和后备力量建设。加强国防动员和民兵预备役工作，抓好国防教育和征兵工作，深入开展“双带双促”和“双拥”共建活动，推动军民融合深度发展，争创全省“双拥”模范城。

10、坚持稳定为重

深化平安常德建设，坚持民意导向，促进社会公平正义，维护社会大局稳定。

创新社会治理。坚持系统治理、依法治理、综合治理、源头治理，改进社会治理方式，促进治理体系和治理能力现代化。以完美社区、美丽乡村建设为基础，推进社会治理重心下移，创新网格化社会管理模式，探索公共服务事项委托办理机制，将更多的人、财、物集中到乡村组织。完善立体化社会治安防控体系，抓好流动人口和特殊人群管理服务，严密防范和打击各类违法犯罪活动。鼓励和支持社会组织参与社会治理。重视网络舆情，依法加强网络社会管理。

维护社会稳定。强化和落实维稳工作责任，对引发重大涉稳问题的单位和个人实行严格追责，对各类违法行为实行依法查处。创新有效预防和化解社会矛盾体制，健全重大事项社会稳定风险评估机制，从源头上、根本上、基础上预防和化解社会矛盾，减少涉稳事件发生。坚持运用法治思维规范信访工作，加强基层基础建设，建立及时就地解决群众合理诉求机制。抓好“六五”普法，做好社区矫正工作，促进人民调解、行政调解、司法调解“三调联动”。

保障公共安全。严格落实安全生产责任，抓好企业安全生产标准化建设、规范化管理，深入开展道路和水上交通、矿山、烟花爆竹、特种设备、建筑施工、消防等领域安全专项整治，持续开展安全生产打非治违行动，开展隐患排查整治，坚决防范重特大安全事故发生。完善食品药品安全监管网络，加大全程监管和风险防控力度，突出热点问题专项治理，推进食用油放心工程，用最严谨的标准、最严格的监管、最严厉的处罚、最严肃的问责，确保人民群众“舌尖上的安全”。强化应急管理，完善自然灾害、事故灾难的监测预警和应急处置机制，提高救援和处置能力。

三、攻坚克难，政府自身建设责任重大

习总书记最近引用过一幅古代的对联：“吃百姓之饭，穿百姓之衣，莫以百姓可欺，自己也是百姓；得一官不荣，失一官不辱，勿说一官无用，地方全靠一官”，直白而又深刻地道出了地方政府在一方发展中的重大责任。在新的历史条件下，我们唯有以最大的决心和勇气、最实的举措和行动，坚持不懈地抓好政府自身建设，才能不辱使命、不废职守。

1、要爱民为民。以开展党的群众路线教育实践活动为契机，忠实履行人民政府为人民的天职。尊群众为主人，拜群众为老师，和群众交朋友，把群众当亲人，决不能把全心全意为人民服务变成三心二意、虚情假意，甚至无情无义。永远做人民群众的亲人、家人、贴心人，关心群众冷暖，体察民生艰难，真心实意办实事、办好事，以干部的“辛苦指数”提升人民群众的幸福指数。

2、要放权限权。要处理好政府与市场、政府与社会、上级与基层的关系。切实转变政府职能，深化行政体制特别是行政审批制度改革，推行地方政府及其工作部门权力清单制度，创新行政管理方式，进一步向市场放权、向社会放权、向基层放权。市政府常务会议已经研究通过，市一级审批事项再压减107项，只保留154项，从今年起执行，这在全省市州中是最少的。区县市要切实把放下去的管理事项接住、办好。要处理好“有限政府”和“有为政府”的关系。全面正确地履行政府职责，避免政府这只“有形的手”变成“闲不住的手”，扎扎实实把地方政府应该加强的公共服务、市场监管、社会管理、环境保护等职责履行好。要更多地推广政府购买公共服务。

3、要依法守法。各级政府要带头维护宪法和法律权威，强化法治思维。要切实做到依法决策，完善重大决策程序和责任追究机制。要严格公正执法，从实体到程序、从内容到形式、从过程到结果都必须符合法律规定。要自觉执行人大及其常委会的决议决定，政府重大决策出台前向人大报告，严格执行规范性文件备案审查和“三统一”制度，接受人大及其常委会的法律监督和工作监督。支持和保障人民政协履行政治协商、民主监督、参政议政职能。认真办理人大代表建议和政协委员提案，高度重视社情民意，虚心听取和采纳各民主党派、工商联、无党派人士的意见建议。接受新闻舆论和社会公众监督。加强监察、审计等内部监督，严格行政问责。

4、要提速提效。要大力提高办事时效，优化办事流程，把办事要件一次告知、一门受理、全程代理、并联审批、超时默认、首问负责、“两集中三到位”等一系列行之有效的制度，在政府各个部门全面推行，决不允许“门好进、话好听、脸好看、事难办”的现象出现。要大力整治发展环境，严格执行“六个严禁、三个一律”规定，着重治理涉企“三乱”和涉项“三强”现象，维护纳税人、企业和投资者的合法权益；整顿规范市场秩序，重点对项目中介服务机构收费贵、服务差、垄断经营等问题进行综合治理，让破坏建设环境、扰乱经济秩序的企业和个人退出常德市场。要大力优化服务平台，加强和完善政务服务中心平台、社区服务平台、社会求助服务平台、电子政务平台和公共资源交易平台建设，积极推行网上办事，打造“不下班的政府”。

5、要谋事干事。“不干，半点马克思主义也没有”。千招万招，不抓落实等于白招；千条万条，不抓落实等于白条。各级政府要强化担当意识、增强担当勇气、提升担当能力，敢于担责、敢于担难、敢于担险。市委作出的决策部署，一定要雷厉风行、坚决执行。市政府抓的事情，一定要议立决、决立行，不争论、不折腾，脚踏实地地干，实事求是地做，以坐不住、等不起的状态，以只争朝夕、不舍昼夜的精神干事创业，坚决摒弃以会议落实会议，以文件贯彻文件，以形式主义反对形式主义的官场陋习。要强化激励和约束，让想干事、能干事、干成事的人有为有位，受到激励和表彰；让不作为、缓作为、乱作为的人没有市场，受到约束和惩戒。

6、要清正清白。想干事是境界，会干事是水平，干成事是能力，不出事是底线。“一辈子的饭不能一餐吃了”。要坚持从严治政，严格落实党风廉政建设责任制，执行好廉洁从政各项规定，把权力关进制度的笼子。要让人民过上好日子，政府就要过紧日子。要坚决贯彻《党政机关厉行节约反对浪费条例》等规定，从严从紧控制“三公”经费，进一步降低行政运行成本。阳光是最好的防腐剂。大力推行公开晒权、公开承诺、公开问政、公开测评、公开曝光，让政府工作阳光操作、阳光运行。全体政府工作人员都要做到政治上跟党走，经济上莫伸手，作风上不丢丑，守住底线，不越红线，不触高压线。对违法违纪的人和事，要坚决查处，决不姑息。

各位代表!

时代创造机遇，实干成就伟业。让我们在市委的坚强领导下，同心同德，锐意进取，攻坚克难，共同描绘新常德新创业的美好画卷，共同书写中国梦的常德篇章!
